# PRECLINICAL DEMENTIA CARE AND PREVENTION IN CONTEXT: FROM INSIGHTS TO ACTION

**DOI:** 10.1093/geroni/igac059.1578

**Published:** 2022-12-20

**Authors:** Daniel Rong Yao Gan, Patricia Heyn, Emily Greenfield

## Abstract

Dementia affects people with lower socioeconomic statuses disproportionately. People with undiagnosed Alzheimer’s disease may or may not express dementia symptoms depending on their brain reserve, cognitive reserve, and their ability to compensate for brain pathology. Much of these depend on their childhood education, but late-life social engagement may also play a role. As lifespan increases globally, preclinical dementia care and prevention in community settings will be increasingly important. The characteristics of everyday environments that are kind to various changes amid declining cognition may enhance the quality of life of older adults with and without dementia. Such environments may be available or unavailable in diverse communities of varying socioeconomic statuses. This collaborative symposium between the ADRD and CEnR interest groups brings together multidisciplinary scholars to imagine psychosocial interventions that could mitigate place-based disparities in cognitive health. The first presentation frames the importance of relational perspectives in a biopsychosocial-ecological model of care amid cognitive decline. The second presentation shows the cognitive impact of networks among kinless older adults across Europe. The third presentation explores community perspectives on preventive brain health programs in various neighborhoods. The fourth presentation centers the voice and action of people with dementia to improve their neighborhood social environments. The final presentation identifies policy implications for community-based care and prevention from network perspectives. Discussions will distil the significance of community for persons living with and without dementia diagnosis to generate place-based interventions that may better support care and prevention in diverse settings, and identify structural barriers to these efforts.

